# Editorial: Innovative OMICs-technology applications to reach a diagnosis and bring new therapies to immune-mediated neurological diseases

**DOI:** 10.3389/fimmu.2023.1348986

**Published:** 2023-12-19

**Authors:** Laura Otero-Ortega, Mireya Fernández-Fournier, Haritz Irizar, David Otaegui, Exuperio Díez-Tejedor

**Affiliations:** ^1^ Neurological Sciences and Cerebrovascular Research Laboratory, Department of Neurology, Neuroscience Area of Hospital La Paz Institute for Health Research-IdiPAZ (La Paz University Hospital, Universidad Autónoma de Madrid), Madrid, Spain; ^2^ Population Health Science and Policy, Icahn School of Medicine at Mount Sinai, New York, NY, United States; ^3^ Multiple Sclerosis Group, Neurosciences Area, Biodonostia Health Research Institute, San Sebastian, Spain

**Keywords:** biomarkers, diagnosis, immune-mediated, nervous system diseases, multiomics, therapeutics

This editorial summarizes the contributions to the Frontiers Research Topic “*Innovative OMICs-technology applications to reach a diagnosis and bring new therapies to immune-mediated neurological diseases*”, established under the *Multiple Sclerosis and Neuroimmunology* section and appearing under the *Frontiers in immunology* and *Frontiers in neurology* journals.

The term immune-mediated neurological diseases refers to a wide spectrum of different neurologic disorders whereby an abnormal immune system response induces damage to certain structures of the nervous system. In addition to this aberrant immunological response, the pathophysiology of these diseases also involves genetic, environmental, microbiome, and potentially other factors.

While an early diagnosis of immune-mediated diseases is often challenging, it is necessary for early treatment initiation. Prompt therapeutic interventions would lead to a decrease in inflammatory activity and, consequently, in the ensuing disability. Given the complexity of these diseases, it is crucial to develop biomarkers for early diagnosis and to shed light on the molecular mechanisms of disease pathogenesis for the identification of new therapeutic targets.

Omic technologies, a set of methodological approaches that allow the interrogation of molecular dimensions at genomic scales, represent a novel approach for the identification of more powerful biomarkers in immune-mediated diseases. This Research Topic aims to shed new light on the latest applications of these technologies in the search for new biomarkers and new therapeutic strategies, as well as in the deepening of our understanding of the molecular mechanisms driving immune-mediated neurological diseases.

We would like to highlight the following contributions:

Verdier et al. present a novel approach based on single-cell cytometry of peripheral blood cells to gain insights into the immune dysregulation underlying the early onset of AChR+ Myasthenia Gravis. In this study, the authors analyze and compare mononuclear cells of 24 patients with AChR+ Myasthenia Gravis (AChr+MG) against those from 16 controls using a cytometer panel consisting of 37 antibodies. The authors find a decrease in monocyte levels and a global decrease of their activity in the AChr+MG group. They also detect an increase in innate lymphoid cells 2 (ILC2s) and CD27- γδ T cells. In this study, single-cell mass cytometry unveils, for the first time, an unexpected dysregulation of innate immune cells in this autoimmune disease predominantly driven by B cells.

Another study, by Amin et al., examined the prevalence in egyptian Multiple Sclerosis (MS) patients for two genetics variants, rs205764 and rs547311, that are located within LINC00513, a long non-coding RNA recently observed to be a positive regulator of the type I IFN pathway. The authors also explored the potential correlation between variation in those two loci and response to several disease modifying therapies. The results indicate that variants at rs205764 are associated with a positive response to fingolimod and with a failure to respond to dimethylfumarate (DMF). Moreover, this polymorphism also showed correlation with disability measured by estimated disability status score. The authors conclude that genetic polymorphisms in this lncRNA may partially drive disease disability and inconsistent responses to treatment in patients with MS.

Adding to the study of mechanisms of treatment response in MS, Ruschil et al., used next generation sequencing of Ig heavy chain transcripts and Ig mass spectrometry to analyze functional changes in B cell populations after cladribine treatment in patients with MS. The authors find that, after cladribine treatment, a decrease of memory B cells and increase of naïve B cells occurs in the peripheral blood of patients. Moreover, the authors observe a significantly decreased number of B cell clones, as well as a significantly lower diversity in the memory B cell subset 6 and 12 months after treatment initiation. The results also revealed a proportion of B cell clones that are maintained during treatment. The authors conclude that the treatment effects of cladribine might be exerted through a reduction of possibly disease relevant clones in the memory B cell subset without disrupting the overall clonal composition.

Finally, Sanchez-Sanz et al. present a study aimed at exploring the immunophenotypic and transcriptomic changes produced by DMF treatment in MS. To that aim, the authors analyze the lymphocyte and monocyte subsets and their gene expression in peripheral blood mononuclear cells of 22 patients with MS at baseline and 12 months after DMF treatment initiation. Their results show an overall decrease in effector (TEM) and central (TCM) memory CD4+ and CD8+ T cells and an increase in CD4+ naïve T cells after DMF treatment. The decrease in TEM cells was greater in responders to treatment, who also showed an increase in NK cells and a resistance to the decrease in intermediate monocytes showed by non-responders. The responder patients also showed significantly different abundances for 3 subpopulations at baseline (NK bright, NK dim and CD8 TCM) and for 4 subpopulations at 12 months (intermediate monocytes, regulatory T cells, CD4 TCM and CD4 TEMRA). At the trascriptomic level, DMF treatment induced a downregulation of pro-inflammatory genes, chemokines, and activators of the NF-kB pathway, with responders showing a higher number of differentially expressed genes compared to non-responders. The authors conclude that patients that respond to DMF treatment exhibit differences in monocyte and lymphocyte subpopulations and a distinct transcriptomic response.

As exemplified by the studies collected in this Research Topic, omics technologies are powerful tools to identify molecular drivers of pathogenesis in all immune-mediate neurological diseases, Multiple Sclerosis and Myasthenia Gravies as examples, as well as to identify biomarkers of treatment response and the corresponding molecular and cellular changes in the immune compartment. The findings generated by these studies and that we have summarized in this editorial, could pave the way for the development of new biomarkers for diagnosis and therapeutic response.

## Author contributions

LO: Writing – original draft. MF-F: Writing – review & editing. HI: Writing – review & editing. DO: Writing – review & editing. ED-T: Writing – review & editing.

